# Annotations

**Published:** 1906-09-22

**Authors:** 


### ANNOTATIONS.

### Vivisection.

The announcement that the King has been pleased to appoint a Royal Commission with Viscount Selby as chairman to inquire into the question of vivisection will be received with feelings of satisfaction by all shades of opinion. The other members include Sir William Church, Sir William Collins, Sir John McFadyean, Dr. W. H. Gaskell, F.R.S., Dr. George Wilson; Mr. Mark Lockwood and Mr. James Tomkinson represent the House of Commons on the Commision; Mr. M. D. Chalmers the Home Office, which issues vivisection licenses; and Mr. A. J. Ram, K.C., the legal element. These names are a guarantee that the question will be examined from all sides, and the Commission's report upon the practice of subjecting live animals to experiments, whether by vivisection or otherwise, and whether any and if so what changes are desirable in the existing laws, together with the result of their inquiries into the present laws and their administration, will be looked forward to with interest. The evidence taken before Mr. Justice Fry's Commission convinced reasonable people that modern hospital practice necessitated the frequent use of the laboratory for the purposes of diagnosis, and that the charges against metropolitan hospitals by ardent anti-vivisectors were not supported by reliable evidence. So much sentiment has been worked up by agitators in regard to vivisection, owing to an unjustifiable confusion of the methods and practices in this and other countries, that the King's action in appointing the present Commission will be cordially welcomed by professional and public opinion. The more closely the question of vivisection is examined into, the more clear must it become that an English gentleman does not cease to exhibit the high character and qualities of his class merely because he devotes his life to scientific research and laboratory work.

### Receiving Houses for Lunatics.

Many people have been long of opinion that the present arrangements for dealing with alleged lunatics are unsatisfactory. What is needed is a receiving house, where the suspected lunatic can be taken and kept under observation for a sufficient time to enable a specialist in lunacy to decide whether or not there is permanent mental derangement. At present, after a few days in the workhouse, where the surroundings may not be such as to soothe the nervous excitement which first brought about the accusation of insanity, the patient is liable to be certified and sent off to an asylum, from which it is not easy to get away. If he were to receive in a preliminary testing-house, where the number of patients would always be small—some being drafted off to the asylums and some sent home—the same skilled attention which is obtainable in the asylum, accompanied by an even sharper scrutiny of his case, there would be a better assurance that when a patient was finally sent to an asylum the case was one which would need at least prolonged treatment, if, indeed, the affliction was not a permanent one. While, on the other hand, a few weeks of careful treatment might restore the balance which was temporarily upset, and enable the patient to go back to his home, as he might leave a hospital, without the formalities necessary to withdrawing from an asylum. Many experienced alienists believe that with prompt treatment in the early stages of the disease incipient insanity could in many cases be checked, and it is likely that the relations, who so often oppose the placing of a patient in an asylum until he has done some grievous mischief, would be more amenable to reason if it were merely a matter of his going to a mental hospital or observation house for proper treatment. The Asylums Committee of the County Council have recommended the Council to promote a Bill in Parliament next year to obtain authority to establish these receiving houses, and it should obtain general support.

### Medical Ethics.

The current number of the "Birmingham Medical Review" contains an important article on medical ethics by Professor Robert Saundby. He lays down the axiom that no undisputed foundation for ethics has been found than the better kind of current opinion in the medical profession. The true ethical attitude, for example, of one man to another is found in the words "whatsoever ye would that men should do to you, do ye even so to them." This apparently simple principle requires a lot of doing in any career, and still more in the medical life. The patients' interests should be the highest consideration. Professor Saundby somewhat vaguely sums up the profession's duty to the State with the words "render unto Cæsar the things that are Cæsar's, and to God the things that are God's." This doctrine of self-abnegation of the medical man either for State purposes or for philanthropic objects is, in our opinion, already carried in this country to its extreme limit. Professor Saundby righteously condemns the odious principle of self-advertisement as tending towards a general lowering of the profession and likely to fill its ranks with sharp business men who, he thinks, would soon elbow out and starve the true scientific worker. On the other hand, he remarks that complaints about advertisement are often childish and based on trifles, and are nearly all the outcome of the odium medicum—a feeling against which all medical men ought to strive. Dr. Dundas Grant, in his speech in his presidential address to the Section of Otology at Toronto, says, "Although a Scotchman, and as such naturally ready to 'improve the occasion,' I am not going to give you a typical 'ethical' lecture. For my part I find that so much of my time and attention is required for the correction of my own shortcomings that I have been unable to occupy myself with those of my neighbours. I would suggest as a good rule to 'mind your own ethics and leave other people's alone.'" We commend this rule for its pawkiness and practical common sense as a good guide to professional life.

